# Advantages of CoS_2_ nano-particles on the corrosion resistance and adhesiveness of epoxy coatings

**DOI:** 10.1038/s41598-024-64429-2

**Published:** 2024-06-26

**Authors:** M. A. Deyab, Majed M. Alghamdi, Adel A. El-Zahhar, Omnia A. A. El-Shamy

**Affiliations:** 1 Egyptian Petroleum Research Institute, Nasr City, Cairo, 11727 Egypt; 2 Department of Chemistry, College of Science, King Khalid University, P.O. Box 9004, 61413 Abha, Saudi Arabia; 3 Research Center for Advanced Materials Science (RCAMS), King Khalid University, Abha, Saudi Arabia

**Keywords:** Anti-corrosion, Coating, CoS_2_, Impedance, Nano-materials, Chemistry, Electrochemistry

## Abstract

Researchers face significant challenges because of the inadequate corrosion resistance and weak adherence of epoxy (EP) coatings. We deal with these issues here by means of a novel nano-composite coating (EP/nano-CoS_2_). In order to create a composite coating (EP/nano-CoS_2_), CoS_2_ nanoparticles (nano-CoS_2_) were prepared and incorporated to an epoxy (EP) resin. The synthesized CoS_2_ was characterized using XRD and FT-IR spectroscopic techniques. The mean particle size was determined using Scherer equation and found to be 19.38 nm. The zeta potential was also determined (− 9.78 mV). Electrochemical impedance spectroscopies (EIS) as well as pull-off assessments were used to quantify the EP/nano-CoS_2_ coating’s anti-corrosion capabilities and adhesive power. The findings demonstrate that the EIS variables of the EP/nano-CoS_2_ composite coating are markedly improved when compared to raw EP coating. The corrosion resistance or even adhesion of EP protective layer can be markedly increased by using the synthesized nanoparticles as nano-fillers.

## Introduction

Despite numerous surface protection approaches^1,2^, the creation of epoxy coatings has recently gained a lot of popularity^3^. Regrettably, by gaining access to the coating's defective areas, corrosive substances like oxygen and aggressive ions could destroy the metal surface^4^.

Nano-materials have gained significant attention in the development of anti-corrosion epoxy coatings due to their unique properties and potential benefits^5,6^. Nano-materials, such as nanoparticles or nanoclays, can be incorporated into epoxy coatings to create a highly effective barrier against corrosive agents^7,8^. The small size and high surface area of nano-materials enable them to form a dense and uniform protective layer, hindering the penetration of moisture, oxygen, and corrosive chemicals to the underlying substrate^9,10^.

Certain nano-materials possess self-healing capabilities, allowing them to repair any damage or defects in the coating. For example, nanocapsules or nanotubes filled with corrosion inhibitors can release their contents when the coating is damaged, effectively preventing further corrosion and extending the coating's lifespan^11,12^. Nanomaterials can improve the adhesion between the epoxy coating and the substrate. By modifying the surface properties of the nanomaterials or introducing functional groups, they can promote stronger interfacial bonding, reducing the risk of delamination or detachment of the coating^13,14^. Nanomaterials can act as active corrosion inhibitors within the epoxy matrix. For instance, nanoparticles like zinc oxide (ZnO), cerium oxide (CeO_2_), or graphene oxide (GO) can release corrosion-inhibiting ions or form a protective layer on the substrate, effectively slowing down the corrosion process^15–17^. Some nano-materials possess excellent UV stability, which is crucial for outdoor applications. By incorporating UV-absorbing or UV-reflecting nanoparticles, the epoxy coating can resist UV degradation, preventing discoloration, chalking, or loss of mechanical properties^18^. Nano-materials can enhance the mechanical properties of epoxy coatings, such as hardness, toughness, and abrasion resistance. Reinforcing nanofillers like carbon nanotubes (CNTs) or nanofibers can provide additional strength and durability to the coating, making it more resistant to physical damage^19^.

Nano-materials offer promising opportunities for developing advanced anti-corrosion epoxy coatings with improved performance, longevity, and environmental sustainability. Ongoing research and technological advancements in this field continue to expand the possibilities for utilizing nano-materials in corrosion protection applications.

Popular findings in the publications showed that nano-fillers could be used to fill the characteristic defects in the epoxy layer. For instance, based on an investigation by Othman et al.^20^, introducing an innovative method of enhancing water barrier and corrosion resistance capabilities by dispersing graphene oxide sheets in the epoxy coating by using the stable surface property of zinc oxide. Because the existence of nano-fillers may obstruct the electrolytes’/ions’ routes for transfer in the EP structure, the coating protective effectiveness can be greatly enhanced^21–23^. Majiidi et al.^24^ created tetragonal GO-ZnO-chitosan coatings to prevent mild steel substances from corroding.

One essential group of transition metals is cobalt sulfides, which are utilized in catalysts, lithium-ion batteries, supercapacitors, magnetic materials, and alkaline rechargeable batteries. The application of cobalt sulfide nanoparticles as coating materials remains challenge. As a result, takes into account for the first time the impacts of nano-CoS_2_ on the adhesion power and corrosion resistant of epoxy coatings.

## Experimental details

### Chemicals and materials

CoCl_2_.7H_2_O, sodium sulfide, ethyl alcohol were supplied from Alpha chemical company. Bisphenol A Epoxy Resins (PE) (Specifications: EEW = 182–192 (gr/eq), concentration (% w/w) = 100%) and polyamine epoxy hardener (content: > 90 wt%) (They were purchased from Egy-coating Company). The 3.5 wt% sodium chloride solution has been produced by diluting AR type 99.8% NaCl using a solution containing distilled water. The substrate was carbon steel pieces (sizes = 4.3 cm × 2.5 cm × 0.02 cm) having the corresponding composition (wt%): 0.08C; 0.5Mn; 0.006P; 0.03Cu; 0.015Cr; 0.012Ni; 0.06Si and balanced Fe. Before applying coatings, the carbon steel samples were polished with SiC paper (degrees = 400–1200) and cleaned with acetone and distilled water, respectively.

### Cobalt sulfide nanoparticles synthesis

Cobalt disulfide nanoparticles were prepared by dissolving 2.379 g (0.01 mol) of CoCl_2_·7H_2_O in de-ionized water. In other flask, 0.02 mol of sodium sulfide was prepared. Then, sulfide solution was added slowly to the cobalt solution during stirring and the reaction completed at 80 °C. The previous mixture was turned gradually to black. The final CoS_2_ precipitate was washed several times using ethyl alcohol. The obtained CoS_2_ nanoparticles was dried at 50 °C then characterized.

### Composite coating (EP/nano-CoS_2_) preparation

The nano-CoS_2_ particles were added to the dimethylformamide (DMF) and the mixture was sonicated for 60 min to improve the CoS_2_ particles dispersion in the EP. The nano-CoS_2_ particles (1.0% by total weight of EP and hardener) were introduced to the EP immediately, with the stirring mechanism running around 1800 rpm for 120 min. Polyamine hardener with balanced ratios has been added to the mixture. The viscosity was adjusted using butyl acetate.

Utilizing the dipping process, EP/nano-CoS_2_ nanocomposite coatings have been put on clean carbon steel substrates. The coated carbon steel specimens were left to cure over 1.0 h around 393 K. Hand carried micrometre (B.C. Ames Company.) was employed to determine the dry layer thickness of the coating. It had a value of 30 ± 5 μm.

### Characterizations tools

The crystalline information of nano-CoS_2_ was obtained using X-ray powder diffractometer (XRD) (PanalyticalXPERT-PRO MPD—Netherlands).

A spectrum of Fourier Transform Infrared Spectroscopy (FTIR) (Perkin Elmer, USA) was employed for the identification of nano-CoS_2_.

The Zeta Potential distribution of CoS_2_ was investigated using Malvern Zetasizer ZS-HT, UK.

### Electrochemical and mechanical tests

Gamry Reference 3000TM using open circuit potential (OCP) with voltage intensity 10 mV within the frequency region 1 Hz–100 kHz was used for the EIS investigations. For EIS data fitting, the Z-View-programme software was employed.

The electrochemical equivalent circuit (EEC) can be done by fitting the impedance data obtained from experiments to the circuit model. Z-View software can import ASCII text files generated by electrochemical stations and perform efficient EIS fits.

The fitted parameters will provide insight into the electrical behavior of the anti-corrosion coating system. It is important to validate the selected circuit model by comparing the modeled impedance response with experimental data not used during parameter estimation. This validation step helps ensure that the chosen circuit model accurately represents the electrical behavior of the anti-corrosion coating system and can be relied upon for further analysis.

The EIS test includes a Pt wire (counter electrode), a coated steel surface (working electrode), a saturated calomel electrode (SCE) (reference electrode), and a 3.5 wt percent sodium chloride (corrosive solution)^25,26^. The ASTM D4541 procedure was utilized to assess the adhesive strength of coatings employing a pull-off adhesion device (GM01-6.3 kN). A perpendicular pulling force is applied to a coating and substrate in pull-off adhesion examination. Both the coating and the metal surface must be cleaned before beginning the test. After that, the glue is ready and put on the metal surface, which is then attached to the covered surface. After then, the actuator of the machine is positioned over the coated surface, and pressure is exerted until the adhesion breaks.

As in prior studies, EP/(1.0%)nano-CoS_2_ coating preparation, Pull-off adhesion test and EIS analyses were conducted out^27–29^.

## Results and discussion

### Characterization

**Figure 1:**
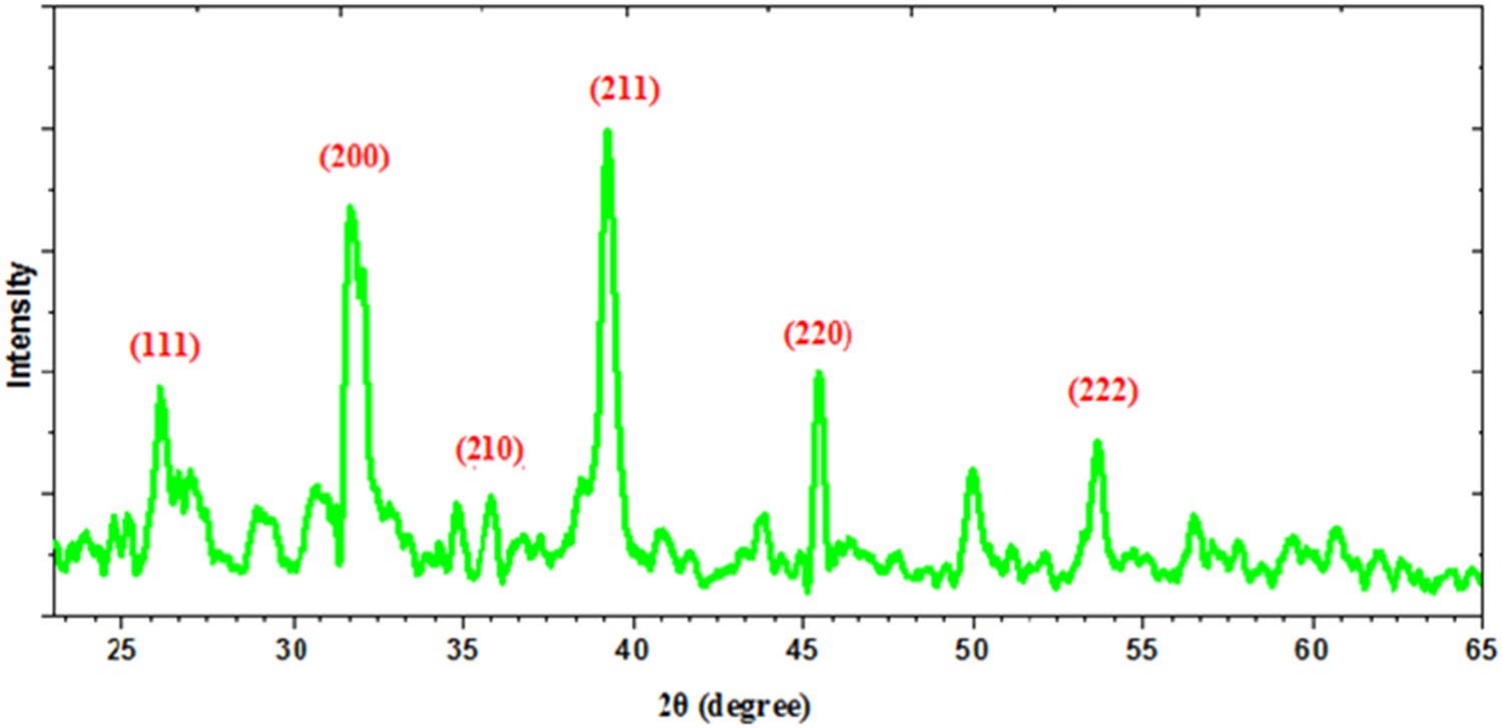
XRD spectra of CoS_2_ nanoparticles.

Figure 1 displays the XRD pattern of the synthesized cobalt sulfide nanoparticles. This pattern indicates that all diffraction lines relate to CoS_2_ in the cubic phase^30^. The peaks located at 26.16°, 31.70°, 35.92°, 39.24°, 45.42°, and 53.67° are corresponding to indices (111), (200), (210), (211), (220), and (222), respectively. The nano-scale of the formed cobalt sulfide nanoparticles confirmed by the broadening of the peaks (see Fig. 1). This is indicating that the synthesis of pure CoS_2_ nanostructures was successful.

Debye-Scherer’s equation (*D* = (0.94λ/*β* cos *ɵ*) was used to calculate the mean particle size (D)^31^ of the synthesized CoS_2_.

Depending on the most sharp, and intense peak (i.e. 211) to substitute in Scherrer equation by using the value of β (the full width at half-maximum value (FWHM) in radians of XRD diffraction lines, the mean particle size of CoS_2_ nanoparticles is 19.38 nm.

**Figure 2:**
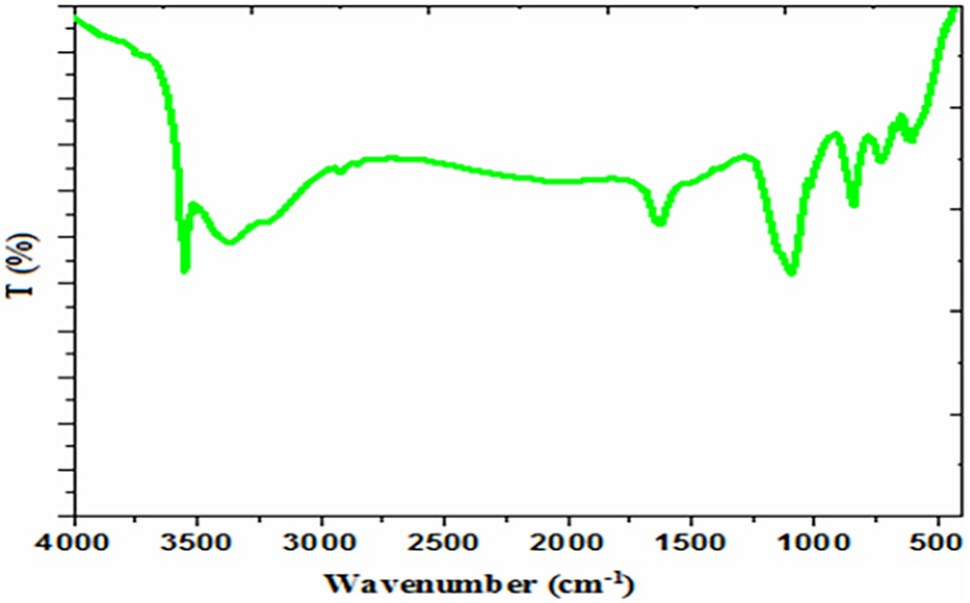
FT-IR spectra of CoS_2_ nanoparticles.

FT-IR measurements were used as another technique to confirm the chemical structure of the synthesized CoS_2_ nanoparticles. Concerning Fig. 2, absorbance peaks appear at 3550.72 cm^−1^ and 1623.63 cm^−1^ are assigned to stretching vibration mode of the hydroxide groups that absorbed on the sulfide surface^30^. The characteristic bands related to sulfides are present at 1095.66 cm^−1^ and 671.36 cm^−1^ due to asymmetric and stretching modes, respectively^32^. Additionally, a small peak at 604.64 cm^−1^ is assigned to the stretching vibrations of the cobalt^33^.

**Figure 3:**
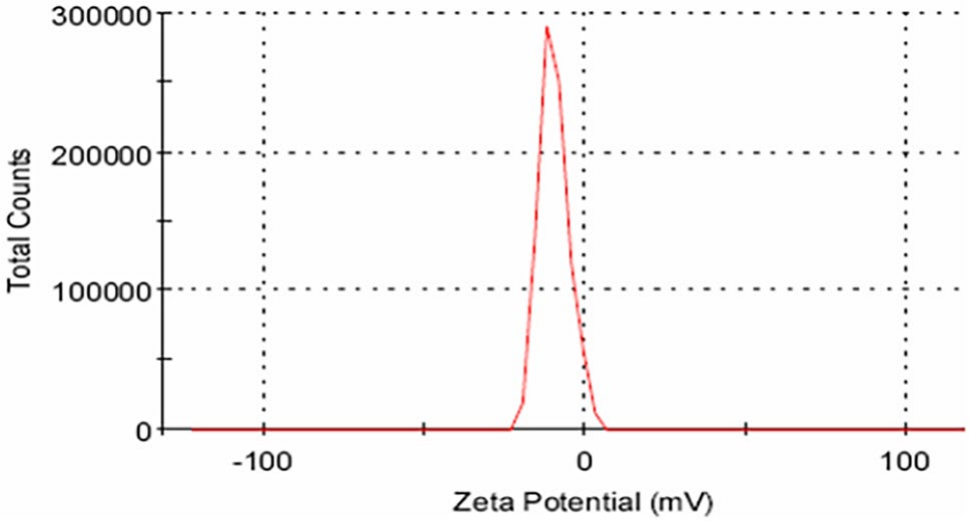
Zeta potential distribution of CoS_2_.

The surface electric charges of the particles are reflected by the zeta potential. Zeta potential is a measure of how strongly charged particles repel one another through electrostatic forces^34^. The resistance of the particles to aggregation in a dispersion system is shown by a high degree of zeta potential, either positive or negative, and this suggests an apparent stability of the system^35^. The value of zeta potential of CoS_2_ nanoparticles was − 9.78 mV indicating good stability (see Fig. 3).

**Figure 4:**
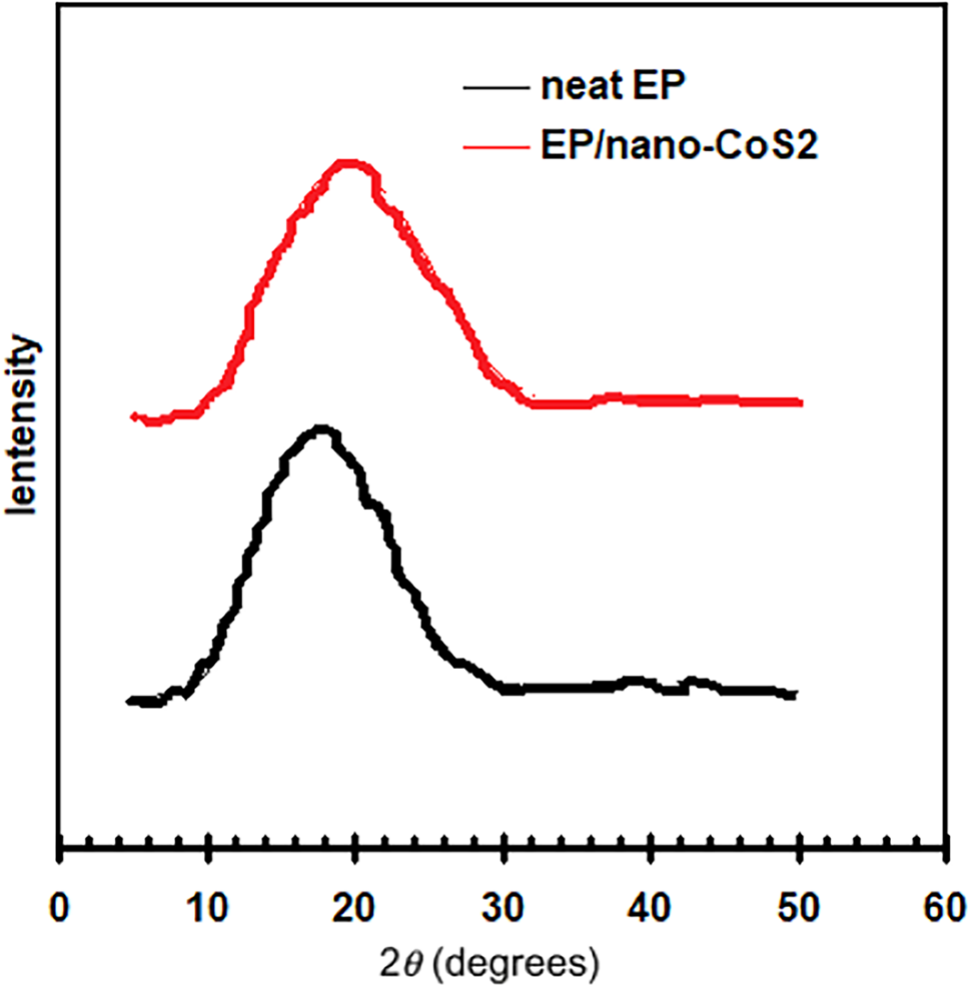
XRD diagrams of neat EP and EP/nano-CoS_2_.

Compare the XRD results of the neat EP and EP/nano-CoS_2_ to understand the impact of nanoparticle incorporation on the material’s crystallographic properties. The XRD diagrams of neat EP and EP/nano-CoS_2_ are shown in Fig. 4. Broad, featureless peaks are usually visible when looking at the XRD pattern of neat EP. This is because the epoxy resin that is usually employed in coatings is amorphous. According to Fig. 4, the diffraction peak of the EP/nano-CoS_2_ vary negligible when compared to those of neat EP, suggesting that the CoS_2_ particles have minimal influence on the structure of EP resin.

### EP/nano-CoS_2_ coating corrosion protection characteristics

**Figure 5:**
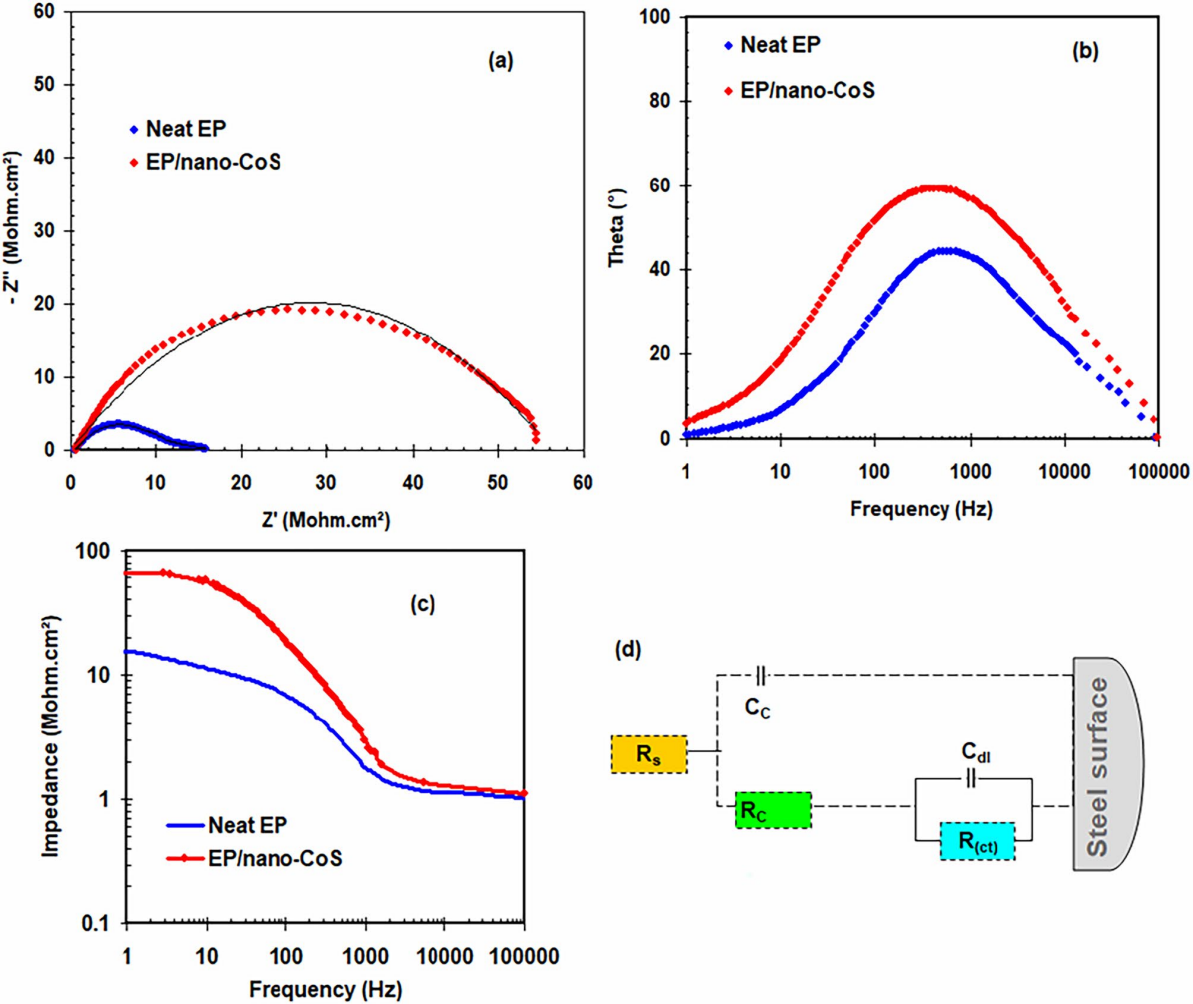
Impedance spectra for carbon steel substrates protected with neat EP and EP/nano-CoS_2_ coating dipped in 3.5% NaCl liquid at 303 K include (**a**) Nyquist, (**b**) Bode-phase angle plots, (**c**) Bode-Impedance, (**d**) EEC of neat EP and EP/nano-CoS_2_ coating.

The corrosion performance of nano-composite coatings that included CoS_2_ nanoparticles was evaluated using EIS estimation. Upon 2 days of soaking in 3.5% NaCl liquid, the Nyquist (Fig. 5a), phase charts (Fig. 5b) and Bode-Impedance curves (Fig. 5c) of neat EP-coated steel and an EP/nano-CoS_2_ coating nano-composite are shown.

As shown in Fig. 5a, the neat EP-coated carbon steel provides a twice constant inside the Nyquist plots. The behavior is caused by the permeability of the epoxy resin coating^36^. The first time constant semicircle was attributed to the epoxy layer's impedance (@high frequency)^36^. The peaks within the intermediate frequency range (10^1^–10^3^ Hz) indicate the pore blockage of corrosive substances in defective areas and the formation process of new coatings^37^. The electrochemical equivalent circuit (EEC) used to assess the apparent impedance variables for neat PE includes the electrolyte-resistance (R_s_), coating-resistance (R_c_), charge-transfer-resistance (R_ct_), coating-capacitance (C_c_), and double-layer-capacitance (C_dl_) (see Fig. 5d). Because of the heterogeneity of coating and double layer formed at the underlying surface, CPE_C_ and CPE_dl_ have to be used instead of the C_c_ and C_dl_ parameters, respectively.

The R_c_ and C_c_ values for neat EP are 10.13 Mohm cm^2^ and 1.2 × 10^–8^ F cm^−2^, respectively. For the same neat EP, the R_ct_ and C_dl_ values are 3.2 Mohm cm^2^ and 4.6 × 10^–8^ F cm^−2^, respectively. This suggests that the neat epoxy layer appears to possess a low level of corrosion impedance.

The substrate in this case exhibits unusual signs of the very corrosive effects. On steel surfaces, chloride ions speed up general corrosion and produce pitting corrosion^38^. Coating debonding is brought on by the creation of corrosion products from the anodic process and the release of hydrogen gas at the cathodic process^39,40^.

In the EP/nano-CoS_2_ coating -coated steel situation (see Fig. 5a–c), quite a semicircle is observed in addition to an increment in R_c_ to 51.6 Mohm cm^2^ and a reduction in C_c_ level to 3.2 × 10^–10^ F cm^−2^. The comparable EEC for steel with the EP/nano-CoS_2_ coating is shown in Fig. 5d. An EP/nano-CoS_2_ coating showed a greater phase angle in comparison to a tidy EP coating, implying that it likely be more adaptable and flexible (see Fig. 5b). It can be inferred from this that adding nano-CoS_2_ improves epoxy’s stiffness and anti-corrosion properties. Noticeably, by combining nano-CoS_2_ into the epoxy composite, corrosion of covered carbon steel seemed to be reduced significantly.

Nano-CoS_2_ coatings act as a physical shield, preventing corrosive charged particles from dispersing through the coating and preventing corrosion^41^. Furthermore, the incorporation of Nano-CoS_2_ helps improve the mechanical features of the epoxy coating^42^.

**Figure 6:**
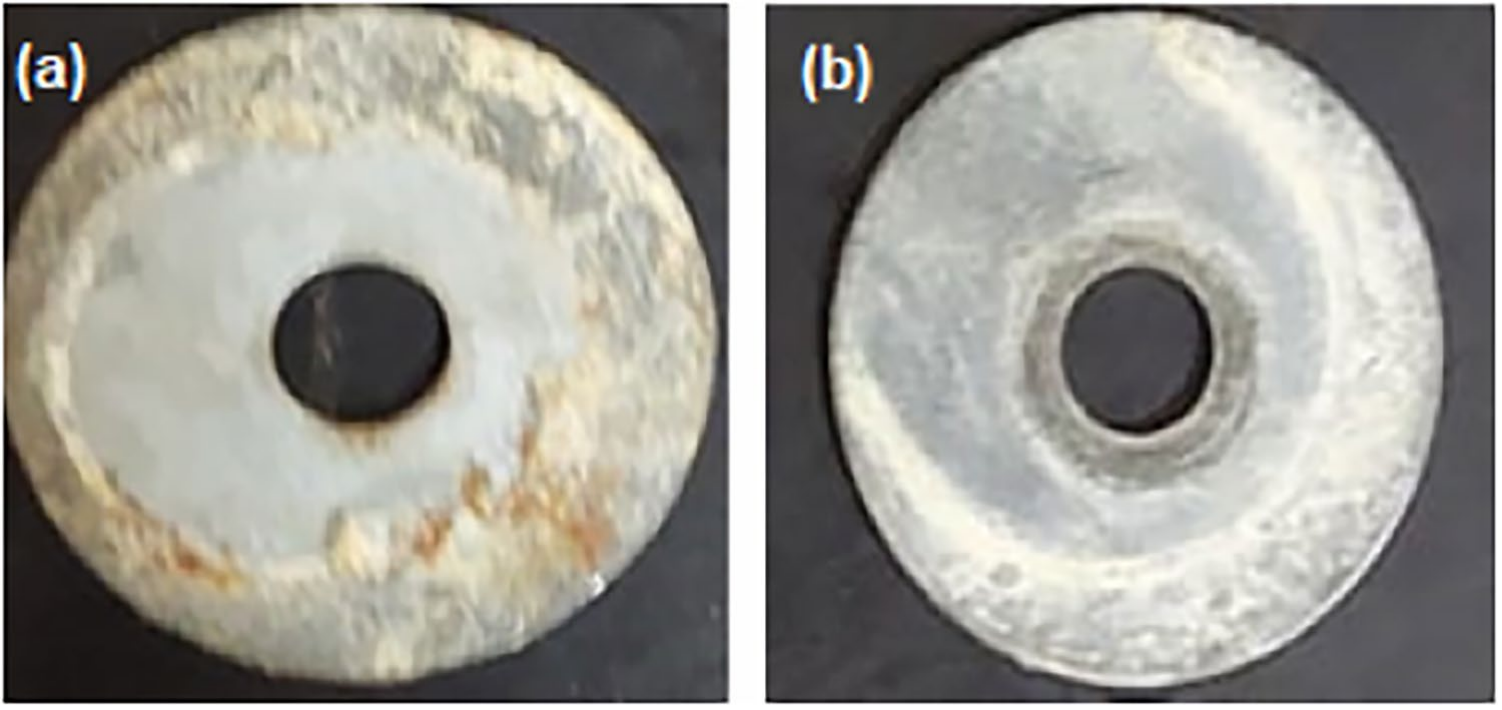
The photographic images of pull-off wet adhesion test in 3.5 wt.% NaCl solution for neat EP (**a**) and EP/nano-CoS_2_ (**b**).

Pull-off tests were used to assess the overall impact of nano-CoS_2_ on the adhesion strength (AS) of EP coating. The photographic images of pull-off wet adhesion test in 3.5 wt.% NaCl solution for neat EP and EP/nano-CoS_2_ are shown in Fig. 6a and b respectively.

The average AS of the neat EP coating (without nano-CoS_2_) became 4.20 MPa, whereas the EP/nano-CoS_2_ coating provided considerably higher values of 12.33 MPa. As a consequence, incorporating CoS_2_ into the EP coating considerably enhanced the coating's adhesion force. This increase in AS could be likened to the restraint of indentation caused by the increased physical interactions between both the EP resin and nano-CoS_2_^43^.

The considerable increase in resistance of the neat EP modified by nano CoS_2_ having a significant specific surface area can be attributed to the following reasons. Actually, nano-CoS_2_ typically fills in voids and pinholes in epoxy coatings, which lowers the cured epoxy coating’s total free volume and raises its cross-linking density^44^. Within the epoxy matrix, nano-CoS_2_ can serve as reinforcing fillers. Enhanced mechanical properties are a result of their high aspect ratio and significant surface area-to-volume ratio. Furthermore, the CoS_2_ nanoparticles may reduce EP dis-aggregation during curing, resulting in a more uniform coating^44^.

## Conclusion

This work is the first to examine the effect of nano-CoS_2_ on the adhesion strength and corrosion resistance of epoxy coatings. The preparation of CoS_2_ nanoparticles (19.38 nm) and their incorporation into an epoxy (EP) resin were done in order to make a composite coating (EP/nano-CoS_2_). In the case of the steel with the EP/nano-CoS_2_ coating, a distorted semicircle is seen, along with an increase in R_c_ to 51.6 Mohm cm^2^ and a decrease in C_c_ level to 3.2 10^–10^ F cm^−2^. While the EP/nano-CoS_2_ coating produced noticeably higher AS values of 12.33 MPa, the average AS of the clean EP coating (without nano-CoS_2_) was 4.20 MPa. The final conclusion demonstrated that using the synthesized nanoparticles as nano-fillers can significantly improve the corrosion resistance or even adhesion of the EP protective layer.

## Data Availability

The datasets used and/or analysed during the current study available from the corresponding author on reasonable request.

## References

[1] El-Shamy OAA (2013). Effectiveness of some nonionic surfactants As corrosion inhibitors for carbon steel in hydrochloric acid solution. Adv. Mater. Res..

[2] Deyab MA, Mele G (2019). Polyaniline/Zn-phthalocyanines nanocomposite for protecting zinc electrode in Zn-air battery. J. Power Sourc..

[3] Deyab MA, De Riccardis A, Mele G (2016). Novel epoxy/metal phthalocyanines nanocomposite coatings for corrosion protection of carbon steel. J. Mol. Liq..

[4] Deyab MA, Abd El-Rehim SS, Hassan HH, Shaltot AM (2020). Impact of rare earth compounds on corrosion of aluminum alloy (AA6061) in the marine water environment. J. Alloys Compounds.

[5] Zehra S, Mobin M, Aslam R (2023). Nanocontainers: A comprehensive review on their application in the stimuli responsive smart functional coatings. Prog. Org. Coat..

[6] Pourhashem S (2020). Polymer/Inorganic nanocomposite coatings with superior corrosion protection performance: A review. J. Ind. Eng. Chem..

[7] Huttunen-Saarivirta E, Vaganov GV, Yudin VE, Vuorinen J (2013). Characterization and corrosion protection properties of epoxy powder coatings containing nanoclays. Progress Org. Coat..

[8] Sari MG, Abdolmaleki M, Rostami M, Ramezanzadeh B (2020). Nanoclay dispersion and colloidal stability improvement in phenol novolac epoxy composite via graphene oxide for the achievement of superior corrosion protection performance. Corrosion Sci..

[9] Behzadnasab M, Mirabedini SM, Esfandeh M (2013). Corrosion protection of steel by epoxy nanocomposite coatings containing various combinations of clay and nanoparticulate zirconia. Corrosion Sci..

[10] Navarchian AH, Joulazadeh M, Karimi F (2014). Investigation of corrosion protection performance of epoxy coatings modified by polyaniline/clay nanocomposites on steel surfaces. Progress Org. Coat..

[11] Akhondi M, Jamalizadeh E (2020). Fabrication of β-cyclodextrin modified halloysite nanocapsules for controlled release of corrosion inhibitors in self-healing epoxy coatings. Progress Org. Coat..

[12] Nazari MH, Zhang Y, Mahmoodi A, Xu G, Yu J, Wu J, Shi X (2022). Nanocomposite organic coatings for corrosion protection of metals: A review of recent advances. Progress Org. Coat..

[13] Randis R, Darmadi DB, Gapsari F, Sonief AA, Akpan ED, Ebenso EE (2023). The potential of nanocomposite-based coatings for corrosion protection of metals: A review. J. Mol. Liquids.

[14] Xavier JR (2022). Novel multilayer epoxy nanocomposite coatings for superior hydrophobic, mechanical and corrosion protection properties of steel. Diamond Relat. Mater..

[15] Ding K, Yuchen Wu, Wei X, Zhu X (2024). Preparation and corrosion resistance mechanism of chromium-free Zn–Al+CeO2 composite coatings. Mater. Chem. Phys..

[16] Nemes PI, Lekka M, Fedrizzi L, Muresan LM (2014). Influence of the electrodeposition current regime on the corrosion resistance of Zn–CeO2 nanocomposite coatings. Surface Coat. Technol..

[17] Kumar AM, Jose J, Hussein MA (2022). Novel polyaniline/chitosan/reduced graphene oxide ternary nanocomposites: Feasible reinforcement in epoxy coatings on mild steel for corrosion protection. Progress Org. Coat..

[18] Najmi P, Keshmiri N, Ramezanzadeh M, Ramezanzadeh B, Arjmand M (2023). Epoxy nanocomposites holding molybdenum disulfide decorated with covalent organic framework: All-in-one coatings featuring thermal. UV-Shielding, Mech. Propert. Compos. Part B: Eng..

[19] Chen S, Wang X, Zhu G, Zhaoxia Lu, Zhang Y, Zhao X, Hou B (2021). Developing multi-wall carbon nanotubes/Fusion-bonded epoxy powder nanocomposite coatings with superior anti-corrosion and mechanical properties. Colloids Surfaces A: Physicochem. Eng. Aspects.

[20] Othman NH, Yahya WZN, Che-Ismail M, Mustapha M (2020). Highly dispersed graphene oxide–zinc oxide nanohybrids in epoxy coating with improved water barrier properties and corrosion resistance. J. Coat. Technol. Res..

[21] Ramezanzadeh B, Haeri Z, Ramezanzadeh M (2016). A facile route of making silica nanoparticles-covered graphene oxide nanohybrids (SiO2-GO); fabrication of SiO2-GO/epoxy composite coating with superior barrier and corrosion protection performance. Chem. Eng. J..

[22] Ma Y (2016). Fabrication of silica-decorated graphene oxide nanohybrids and the properties of composite epoxy coatings research. Appl. Surf. Sci..

[23] Zhu X (2019). In-situ modulation of interactions between polyaniline and graphene oxide films to develop waterborne epoxy anticorrosion coatings. Prog. Org. Coat..

[24] Majidi HJ, Mirzaee A, Jafari SM, Amiri M, Shahrousvand M, Babaei A (2020). Fabrication and characterization of graphene oxide-chitosan-zinc oxide ternary nano-hybrids for the corrosion inhibition of mild steel. Int. J. Biol. Macromol..

[25] El-Katori EE, Nessim MI, Deyab MA, Shalabi K (2021). Electrochemical, XPS and theoretical examination on the corrosion inhibition efficacy of stainless steel via novel imidazolium ionic liquids in acidic solution. J. Mol. Liquids.

[26] Deyab MA, Guibal E (2020). Enhancement of corrosion resistance of the cooling systems in desalination plants by green inhibitor. Sci. Rep..

[27] Deyab MA, Fouda AS, Osman MM, Abdel-Fattah S (2017). Mitigation of acid corrosion on carbon steel by novel pyrazolone derivatives. RSC Adv..

[28] Deyab MA, Słota R, Bloise E, Mele G (2018). Exploring corrosion protection properties of alkyd@lanthanide bis-phthalocyanine nanocomposite coatings. RSC Adv..

[29] Deyab MA, Hamdi N, Lachkar M, El Bali B (2018). Clay/Phosphate/Epoxy nanocomposites for enhanced coating activity towards corrosion resistance. Prog. Org. Coat..

[30] Pourmortazavi SM, Rahimi-Nasrabadi M, Larijani B, Karimi MS, Mirsadeghi S (2018). Electrochemical synthesis of cobalt disulfide nanoparticles and their application as potential photocatalyst. J. Mater. Sci. Mater. Electron..

[31] El-Shamy OAA, Nassar IM, Ragab AA (2022). Novel anticorrosive coatings based on nanocomposites of epoxy, chitosan, and silver. Polym. Bull..

[32] El-Fawal EM, El-Shamy OAA (2019). Photodegradation enhancement of 2-chlorophenol using ZnO–CdS@CS nanocomposite under visible light. Int. J. Environ. Sci. Technol..

[33] Kristl M, Dojer B, Gyergyek S, Kristl J (2017). Synthesis of nickel and cobalt sulfide nanoparticles using a low cost sonochemical method. Heliyon.

[34] Elshamy OA, El-Fawal EM (2021). Synthesis of NiFe 2 O 4 @AC/UiO-66(Zr) for enhancement of the photocatalytic performance of alizarin yellow r under visible-light. ChemistrySelect.

[35] El-Dib FI, Mohamed DE, El-Shamy OAA, Mishrif MR (2020). Study the adsorption properties of magnetite nanoparticles in the presence of different synthesized surfactants for heavy metal ions removal. Egypt. J. Pet..

[36] Deyab MA (2017). Synthesis and characteristics of alkyd resin/M-Porphyrins nanocomposite for corrosion protection application. Prog. Org. Coat..

[37] Cheng L, Liu C, Wu H, Zhao H, Mao F, Wang L (2021). Mussel-inspired delivery system for enhancing self-healing property of epoxy coatings. J. Mater. Sci. Technol..

[38] Deyab MA (2019). Enhancement of corrosion resistance in MSF desalination plants during acid cleaning operation by cationic surfactant. Desalination.

[39] El-Shamy OAA, Deyab MA (2023). Novel anticorrosive coatings based on nanocomposites of epoxy, chitosan, and silver. Mater. Lett..

[40] Chengrong Gu, Junying Hu, Zhong X (2020). The coating delamination mitigation of epoxy coatings by inhibiting the hydrogen evolution reaction. Progress Org. Coat..

[41] Deyab MA, Mele G (2020). Stainless steel bipolar plate coated with polyaniline/Zn-Porphyrin composites coatings for proton exchange membrane fuel cell. Sci. Rep..

[42] El-Shamy OAA, Deyab MA (2023). Improvement of the corrosion resistance of epoxy coatings with the use of a novel zinc oxide-alginate nanoparticles compound. Mater. Lett..

[43] Deyab MA, Awadallah AE (2020). Advanced anticorrosive coatings based on epoxy/functionalized multiwall carbon nanotubes composites. Progress Org. Coat..

[44] Becker O, Varley R, Simon G (2002). Morphology, thermal relaxations and mechanical properties of layered silicate nanocomposites based upon high-functionality epoxy resins. Polymer.

